# Design and Performance of a Focus-Detection System for Use in Laser Micromachining

**DOI:** 10.3390/mi7010002

**Published:** 2016-01-04

**Authors:** Binh Xuan Cao, Munju Bae, Hyonkee Sohn, Jiyeon Choi, Youngduk Kim, Jeng-o Kim, Jiwhan Noh

**Affiliations:** 1 Department of Laser and Electron Beam Application, Korea Institute of Machinery & Materials (KIMM), Daejeon 34103, Korea; xuanbinh.cao@gmail.com (B.X.C.); rlxo175@kimm.re.kr (M.B.); hsohn@kimm.re.kr (H.S.); jchoi@kimm.re.kr (J.C.); jokim@kimm.re.kr (J.K.); 2 Department of Nano-Mechatronics, Korea University of Science and Technology (UST), Daejeon 34113, Korea; 3 Department of Mechanical Engineering, Korea Advanced Institute of Science and Technology (KAIST), Daejeon 34141, Korea; mrcrack@kaist.ac.kr

**Keywords:** detection of focal position, focal position, laser micromachining

## Abstract

We describe a new approach for locating the focal position in laser micromachining. This approach is based on a feedback system that uses a charge-coupled device (CCD) camera, a beam splitter, and a mirror to focus a laser beam on the surface of a work piece. We tested the proposed method for locating the focal position by using Zemax simulations, as well as physically carrying out drilling processes. Compared with conventional methods, this approach is advantageous because: the implementation is simple, the specimen can easily be positioned at the focal position, and the dynamically adjustable scan amplitude and the CCD camera can be used to monitor the laser beam’s profile. The proposed technique will be particularly useful for locating the focal position on any surface in laser micromachining.

## 1. Introduction

In laser ablation processing, carefully maintaining the focal position, *i.e.*, the position associated with the minimum waist diameter, on the work surface is a crucial step in the fabrication process. This ensures that the correct laser power density is used to form a line or a hole on the specimen.

Techniques have been developed to identify the focal position more quickly and precisely. The conventional method uses a guide beam and involves adjusting the specimen’s position to minimize the waist of the beam in z-scan experiments [1,2]. However, this method’s application is limited to simple fabrication processes that do not require high accuracy. Other methods in laser processing involve modifying the specimen in order to find the focal position, including placing the specimen on a worktable and making test spots on the specimen’s surface while adjusting the worktable height or using a measurement beam to detect the electromagnetic field of the beam [3,4,5,6]. Other reports discuss the use of a confocal optical system and other auto focusing systems [7,8,9,10,11,12,13,14] to measure a curved surface. According to these methods, some of which employ an auxiliary laser [11,12] or confocal sensor [14], the measurement data are used to move the focusing lens such that the laser always maintains a focused spot on the curved surface. The surface measurement apparatus associated with the confocal optical system is sensitive to the alignment of the optical components. However, signal processing becomes difficult depending on the type of pulse laser used as the light source in the confocal optical system. Presently, many laser-machining researchers employ ultrafast lasers; however, with these lasers, the capacitor- and resistance-based signal processing clearly show limitations.

In order to overcome these limitations, this paper introduces a feedback system for obtaining the focal position on a measured surface using a mirror, a beam splitter, and a charge-coupled device (CCD) camera. The proposed method for collecting surface data involves the use of an optical system that can maneuver the specimen into the position corresponding to the smallest part of the laser’s spot. Because of its capacity to control the specimen at this spot, the proposed system is called a “focus finder”. The primary advantage of this method is that it reduces the difficulty in accurately identifying the laser’s focal position. The second advantage is that the CCD camera can be used to monitor the laser beam profile. Such monitoring is required because changes in the laser beam’s state lead to changes in the machining quality. However, one disadvantage of using the CCD camera in laser processing is that it is difficult to accurately measure the diameter of the laser beam. In the present study, this disadvantage is overcome by using a laser-power control apparatus.

## 2. Experimental Methods

### 2.1. Concept of Design

**Figure 1:**
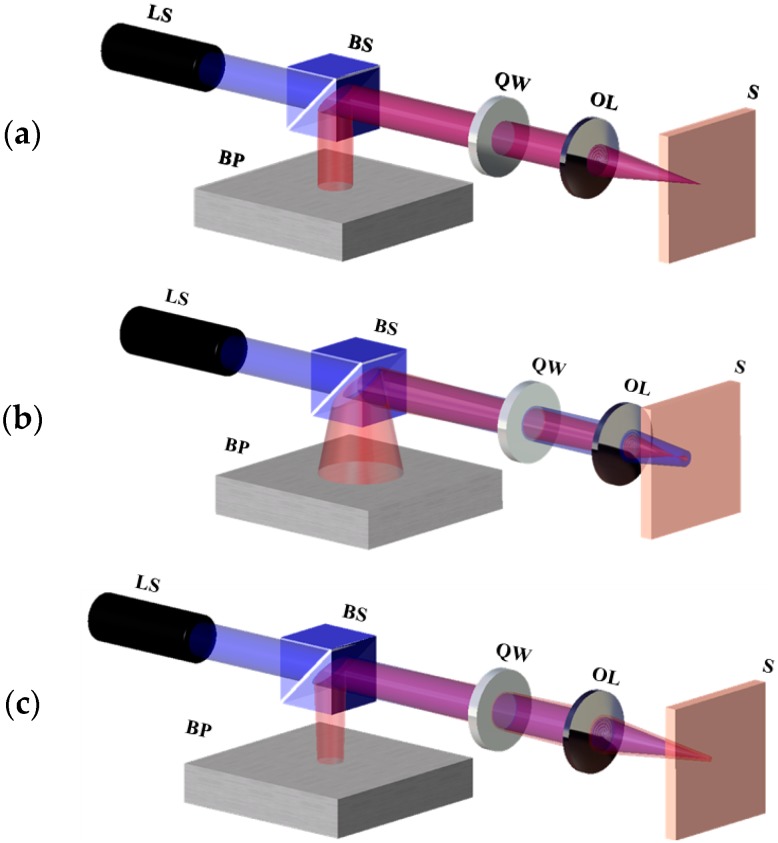
Variation in divergence angle according to specimen’s position. (**a**) Specimen located at the focal position; (**b**) Specimen located in front of the focal position; (**c**) Specimen located behind the focal position. LS: Laser source, BS: Beam splitter, QW: Quarter-wave plate, OL: Objective lens, S: Specimen, BP: Beam profiler.

As shown in Figure 1, the variations in the divergence angle and CCD camera spot size depend on to the specimen’s position. Figure 1a–c show the cases where the specimen is located at the focal position, in front of the focal position, and behind the focal position, respectively. The laser beam passes through the beam splitter, the quarter-wave plate, and the focusing lens before focusing on the specimen. The beam reflected from the specimen passes through the lens and quarter-wave plate to reach the beam splitter, where it is reflected to ultimately arrive at the CCD camera. A silicon wafer was used as the specimen for the beam-profile testing. All of the specimen surfaces were polished before the experiment for the optimal reflection.

Charge-coupled device (CCD) cameras are optical sensors with an array of photodiodes that can measure the beam profile of an arriving beam, and are thus commonly referred to as “beam profilers”. We denote the beam size on the CCD camera as D. As the specimen moves from left to right, the value of D decreases monotonically. As shown in Figure 1a, the specimen is located at the focal position; thus, the reflected rays are parallel and are measured to have a beam size of D = D_focus_ at the CCD camera. In Figure 1b, the specimen is located in front of the focal position; thus, the reflected rays diverge and D is larger than D_focus_. In Figure 1c, the specimen is located behind the focal position; hence, the reflected rays converge and D is measured to be smaller than D_focus_.

**Figure 2:**
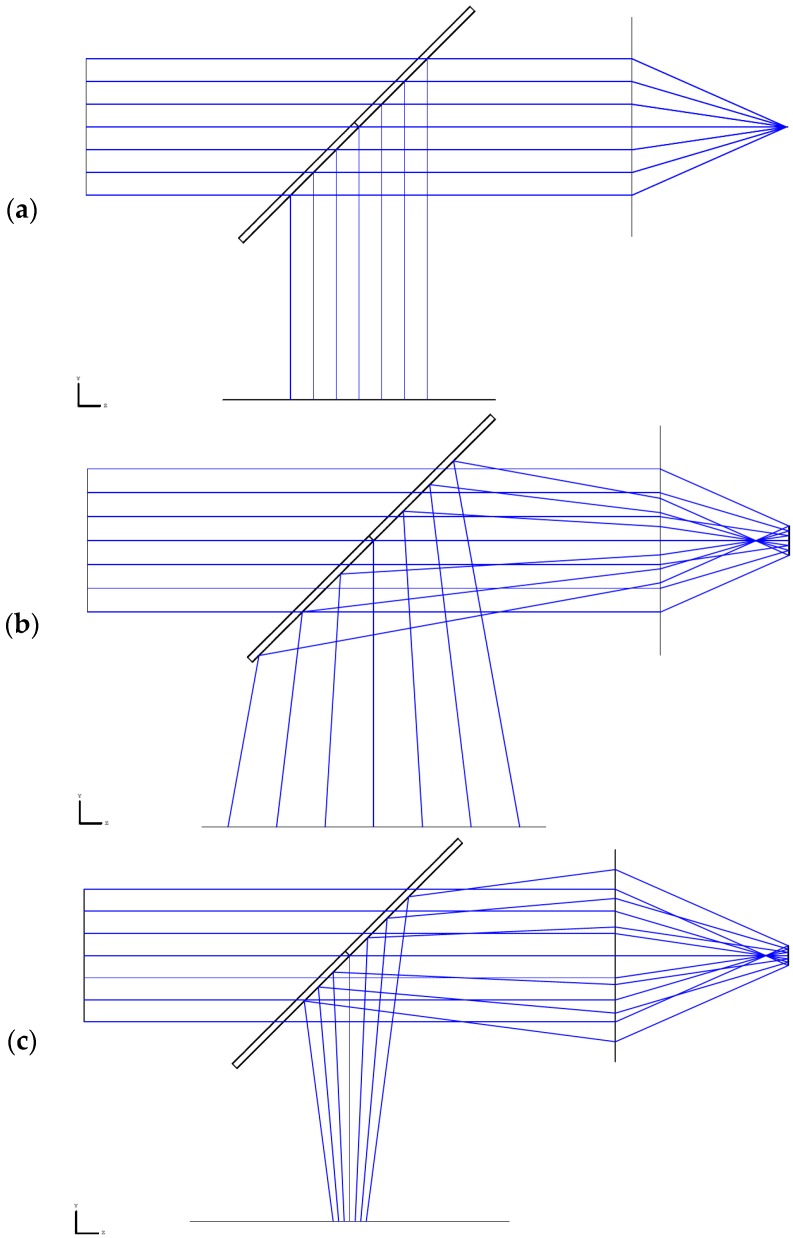
3D layout ray-tracing results according to specimen position. The specimen is located at (**a**) the focal position; (**b**) in front of the focal position; and (**c**) behind the focal position.

Figure 2 shows the ray-tracing results according to the specimen position obtained with Zemax (Zemax LLC, Stansted, United Kingdom). As expected, the reflected rays are parallel when the specimen is located at the focal position, and they diverge when the specimen is in front of the focal position. Furthermore, the converging rays enter the CCD camera when the specimen is located behind the focal position.

### 2.2. Calibration Method for Focus-Finder System

**Figure 3:**
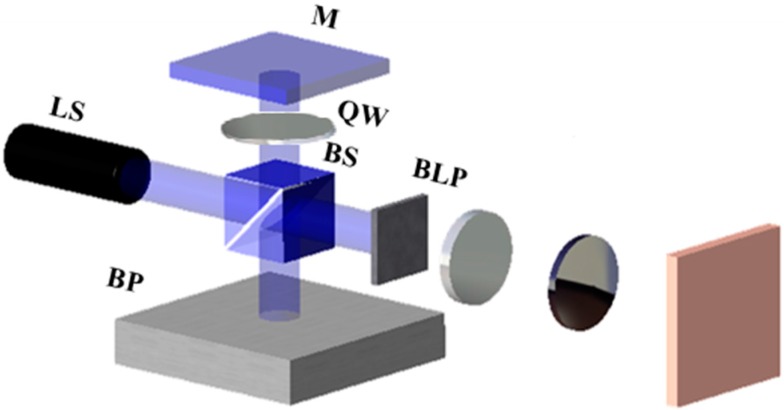
Schematic of the focus finder with an added mirror. BLP: Blocking plate.

We can discover whether the specimen is at the focal position by determining the spot size D_focus_, which is obtained when the specimen is at the focal position. To determine D_focus_, a mirror and a quarter-wave plate are placed in parallel to the beam splitter surface (on the side opposite to the beam profiler), as shown in Figure 3, and a blocking plate is installed at the focusing lens on the specimen side to prevent beam reflection. Thus, some of the beam reflected from the beam splitter is reflected by the quarter-wave plate and mirror. This reflected beam is measured by the CCD camera and inferred to have a beam size of D_focus_. After D_focus_ is measured, the blocking plate, which is currently installed beside the focusing lens, is removed and then reinstalled at the mirror side. At this point, the beam reflected from the specimen is received by the beam profiler. The specimen is moved until the beam size is equal to D_focus_, at which point it becomes known that the specimen is located at the focal position.

**Figure 4:**
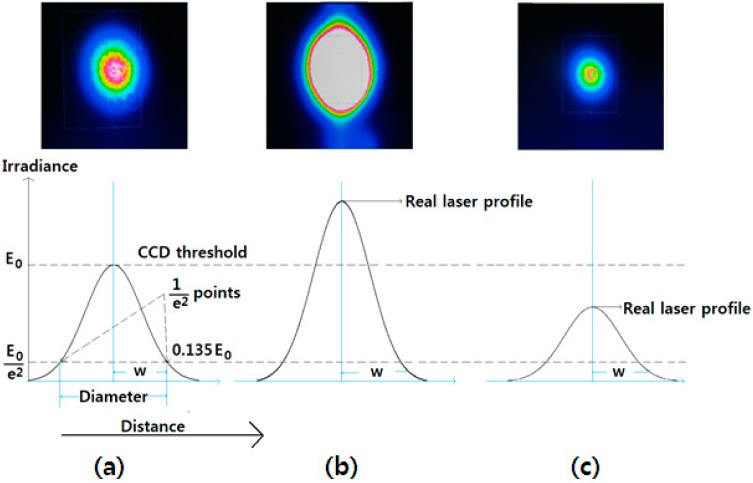
Measurement limitations of the charge-coupled device (CCD) camera. (**a**) Optimized laser power; (**b**) high laser power; and (**c**) low laser power.

One issue that must be considered when locating the focal position using the method described above concerns the inaccuracy of the laser beam measurement when the CCD camera is used. We define spot size as the radius of the beam. Because laser beams are Gaussian, the laser beam size can depend on the laser power. As the laser power increases, the irradiance of the beam decreases gradually at the edges. The distance across the center of the beam for which the irradiance (intensity) equals 1/e^2^ of the maximum irradiance (1/e^2^ = 0.135) is defined as the beam diameter. The spot size (*w*) of the beam is defined as the radial distance from the central point of maximum irradiance to the point at which the irradiance drops by a factor of 1/e^2^. Therefore, in order to accurately measure the laser beam size, the CCD camera must be adjusted to the optimal laser power. The laser beam was initially transmitted over the beam splitter and linearly polarized. After passing over the quarter plate, the beam was circularly polarized. Next, the beam was reflected on the specimen. The circular polarization of the incoming beam is opposite to that of reflected beam. Then the beam was again passed over quarter plate; the polarization phase shift is π in total. Next, the laser beam passed through the beam splitter again and was reflected without any transmission. The entire laser power was thus transmitted to the CCD camera without any loss. Figure 4 shows the limitations of the CCD camera laser beam measurement.

**Figure 5:**
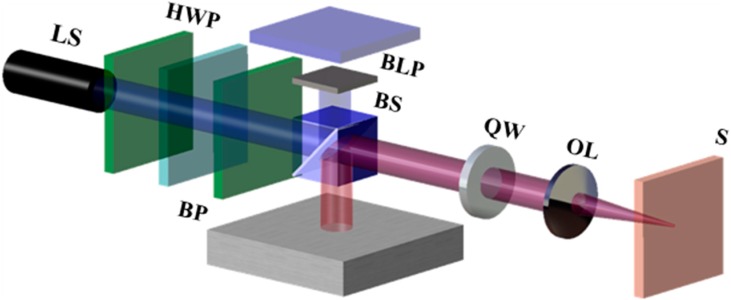
Schematic of the laser power control unit, which employs two polarizers and a half-wave plate (HWP).

Consider three cases of the beam size profile: optimal laser power, laser power greater than the optimal laser power, and laser power lower than the optimal power. Figure 4a–c show images obtained by the CCD camera, along with a graph demonstrating the CCD threshold and the laser profiles for the three cases listed above. The laser beam sizes are 3.084, 3.669, and 1.989 mm in Figure 4a–c, respectively. An SPI laser (G4, 50 W; beam diameter ~3 mm, wavelength 1064 nm, pulse duration 5 ns, *M*^2^ = 1.3, random polarization, SPI Lasers Ltd, Southampton, United Kingdom) was used along with a BeamOn 2/3 CCD camera (Duma Optronics, Nesher, Israel). Furthermore, in order to optimize the laser power, as shown in Figure 4a, we used a laser power control apparatus that included a polarizer, and half-wave plate. The optical components were arranged such that the distance between the beam splitter and the mirror was 100 mm; the distance between the beam splitter and the focusing lens was 300 mm; and the distance between the beam splitter and the beam profiler was 800 mm, as shown in Figure 5. Different spot sizes can be measured according to the laser power, even for the same laser source. This means that the laser beam’s peak value must be adjusted to the CCD camera’s threshold value in order to obtain accurate measurements of the laser beam size.

The main objective of this study is to determine the specimen’s focal position. To this end, we used a microstage to move the specimen. To begin, we picked an arbitrary position and defined it to be the origin of the axis. Next, the focal position required to locate the specimen was determined, and its coordinate was referred to as *l*. The beam that was reflected by the mirror passed through the beam splitter, and was then measured by the beam profiler; this beam was measured to be 1.988 mm in the horizontal direction and 2.222 mm in the vertical direction. The horizontal and vertical dimensions differed because the beam was not an ideal Gaussian beam. The average of the horizontal and vertical dimensions was 2.105 mm, and D_mirror_ was defined to be this value.

## 3. Results and Discussions

**Figure 6:**
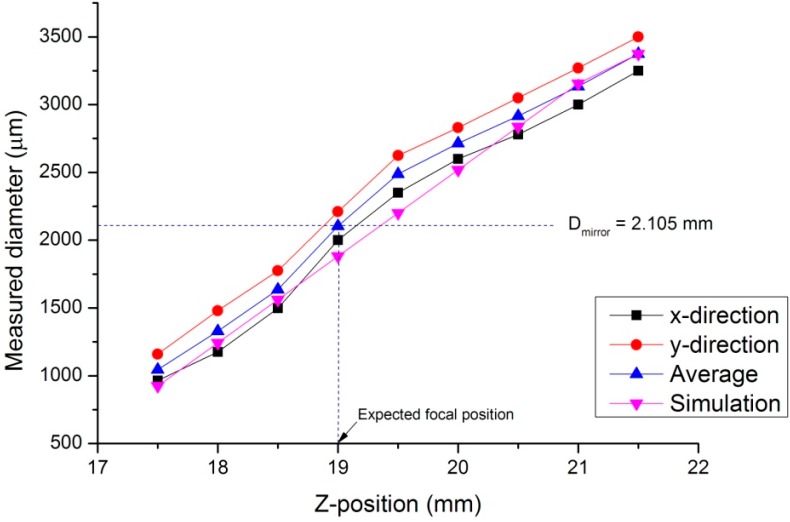
Variation in the beam diameter as measured by the charge-coupled device (CCD) as a function of the specimen’s position. The purple line depicts the optical simulation using Zemax, with the parameters specified in the experiment explained above. The black and red lines indicate the horizontal and vertical beam dimensions, respectively. The blue line indicates the average of the horizontal and vertical beam dimensions.

Figure 6 shows a graph of the beam size as measured by the CCD camera when the specimen was moved along the *z*-direction. Because the absolute position of the specimen is difficult to measure, an arbitrary reference was set, and the distance between the specimen and this reference was measured. This distance is represented by the values on the horizontal axis of Figure 6. Because D_focus_ is 2.105 mm, we can estimate that focusing will occur at the specimen position of *l* ≈ 19 mm.

We measured the average beam diameter in the horizontal direction (*i.e.*, the *x*-direction) and in vertical direction (*i.e.*, the *y*-direction) (blue line). As shown in the figure, a gap exists between the blue line and the purple line (fabrication *vs.* simulation) owing to experimental factors. The purple line (simulation) represents the results under ideal conditions. However, during fabrication, many factors can affect the result. For example, error during the optical alignment process is a crucial factor affecting the overall results. Furthermore, the quality of the specimen’s surface also significantly affects the results. Because the specimen’s surface is not an ideal mirror, the reflected beam is not identical to the incident beam when the beam diameter is measured at the CCD; as a result, what we obtain on the CCD is the actual profile of the reflected beam from the specimen. In this study, we attempted to avoid these factors by improving the optical alignment process and cleaning the specimen’s surface.

**Figure 7:**
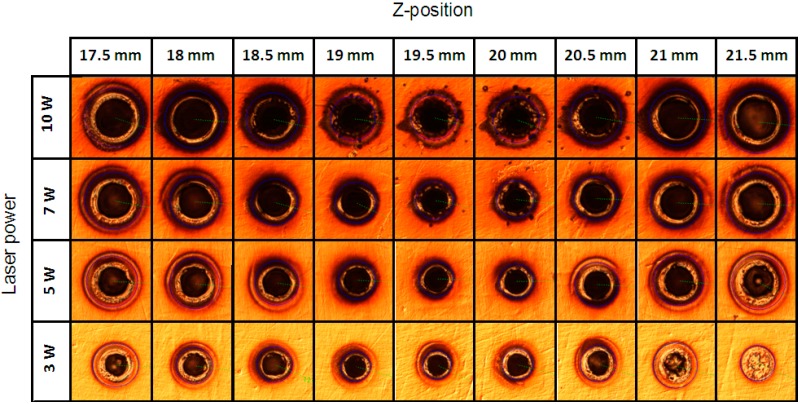
The percussion drilling results for laser beam propagation measurements.

**Figure 8:**
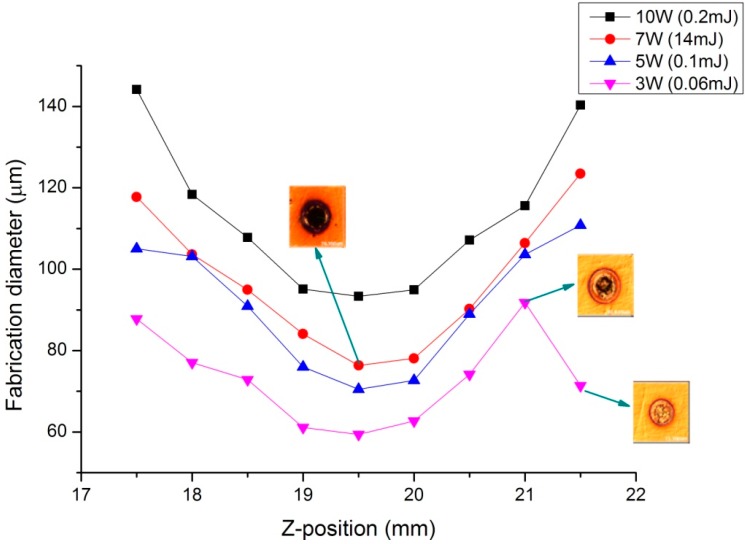
Graph of the percussion drilling results.

Figure 7 and Figure 8 show the images and the graph of fabrication results by percussion drilling when the specimen was moved in the *z*-direction. Figure 7 shows the diameters of the holes marked by the laser fabrication beam on the specimen’s surface around the focal position, as measured through microscope observations (Microscope–Nikon LV150, Nikon, Tochigi, Japan). The laser powers used were 3, 5, 7, and 10 W; the pulse duration was 10 ns; the pulse repetition rate was 50 kHz; the machining time was 0.1 ms; and the focal length of the focusing lens was 80 mm. The smallest fabrication diameter was obtained at the specimen position of *l* = 19.5 mm; therefore, the focal position was located at *l* = 19.5 mm. The focus finder estimated the focal position to be *l* = 19 mm; thus, the error was approximately 0.5 mm.

**Figure 9:**
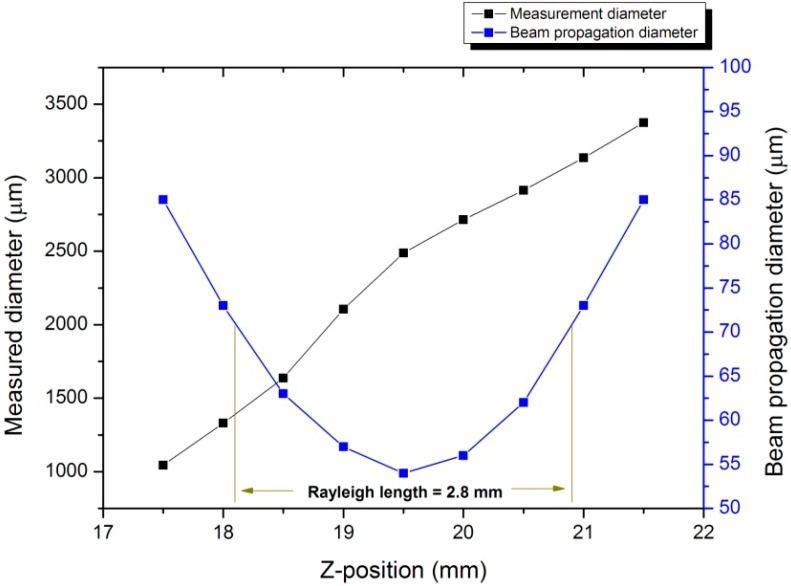
Variation in the propagation diameter as a function of the specimen’s position.

Figure 9 shows the beam size as measured by the CCD camera and the actual laser beam propagation profile when the specimen was moved in the *z*-direction. The blue line in the figure shows the beam propagation profile obtained using the beam size derived from the fabrication diameter. The laser beam diameter at the beam waist in the laser beam profile is 0.054 mm.

The following equation can be used to calculate the Rayleigh length as 2.8 mm:

(1) $$z=\pm \frac{\lambda M^{2}}{\pi }\sqrt{\rho^{2}-1}{\left(\frac{f}{w_{L}}\right)}^{2}$$

where *z* represents the Rayleigh length, ρ represents the beam tolerance, $w_{L}$ represents the entrance beam diameter, and $M^{2}$ represents the beam quality factor. Here, ρ = $\sqrt{2}$, λ = 1064 nm, *M*^2^ = 1.3, *f* = 100 mm, and $w_{L}$ = 3.94 mm. The error of 0.5 mm for the focus finder with a Rayleigh length of 2.8 mm shows that the proposed focus finder is sufficient for use because the power density produced by this system satisfies the required energy density in laser micromachining. Furthermore, according to the formula, the Rayleigh length is proportional to the wavelength of the laser beam and the focal length. Therefore, the Rayleigh length increases with the wavelength and focal length. This means that when the wavelength and focal length increase, our measurements become more accurate because of the increasing ratio between the Rayleigh length and the mismatch of measurements, and *vice versa*.

## 4. Conclusions

A focus-finder system based on the use of a beam splitter, a mirror, and a CCD camera has been demonstrated. We provided a direct experimental comparison with theoretical simulations in order to achieve a better understanding of the underlying mechanisms. We also analyzed the power of the reflected beam and provided a method for obtaining an accurate beam profile with the CCD camera. Compared with the traditional method, the proposed method boasts the following advantages: it is simple, and it yields a highly accurate focal position. This method has potential applications in finding the focal position in all laser micromachining applications, especially those involving arbitrary surfaces.
